# (Acetyl­acetonato-κ^2^ *O*,*O*′)bis­[2-(naphth[1,2-*d*][1,3]oxazol-2-yl)phenyl-κ^2^ *C* ^1^,*N*]iridium(III)

**DOI:** 10.1107/S1600536811034222

**Published:** 2011-08-27

**Authors:** Guojie Yin

**Affiliations:** a Department of Environment Engineering and Chemistry, Luoyang Institute of Science and Technology, 471023 Luoyang, People’s Republic of China

## Abstract

In the crystal structure of the title compound, [Ir(C_17_H_10_NO)_2_(C_5_H_7_O_2_)], the Ir^III^ atom is *O*,*O*′-chelated by the acetyl­acetonate group and *C*,*N*-chelated by the 2-aryl­naphth[1,2-*d*]oxazole groups. The six-coordinate metal atom displays a distorted octa­hedral geometry.

## Related literature

For the synthesis and reactions of some 2-aryl­naphth[1,2-*d*]oxazole derivatives, see: Abbady (1979 ▶). For the synthesis and characterization of phospho­rescent cyclo­metalated iridium complexes, see: Lamansky *et al.* (2001 ▶). 
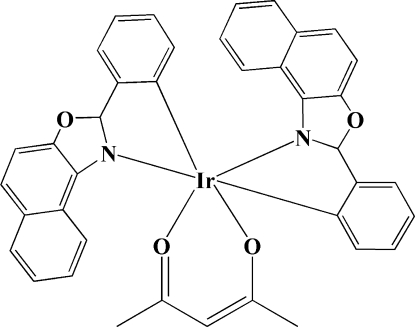


## Experimental

### 

#### Crystal data

[Ir(C_17_H_10_NO)_2_(C_5_H_7_O_2_)]
*M* *_r_* = 779.83
Monoclinic, 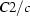

*a* = 16.640 (3) Å
*b* = 17.384 (3) Å
*c* = 21.461 (4) Å
β = 95.026 (2)°
*V* = 6184.3 (19) Å^3^
*Z* = 8
Mo *K*α radiation
μ = 4.36 mm^−1^
*T* = 273 K
0.30 × 0.20 × 0.20 mm

#### Data collection

Bruker SMART CCD area-detector diffractometer
Absorption correction: multi-scan (*SADABS*; Sheldrick, 2004 ▶) *T*_min_ = 0.354, *T*_max_ = 0.476
28178 measured reflections
7558 independent reflections
6091 reflections with *I* > 2σ(*I*)
*R*_int_ = 0.031

#### Refinement

*R*[*F* ^2^ > 2σ(*F* ^2^)] = 0.023
*wR*(*F* ^2^) = 0.051
*S* = 1.01
7558 reflections
415 parameters
H-atom parameters constrained
Δρ_max_ = 0.56 e Å^−3^
Δρ_min_ = −0.31 e Å^−3^

### 

Data collection: *SMART* (Bruker, 2001 ▶); cell refinement: *SAINT* (Bruker, 2001 ▶); data reduction: *SAINT*; program(s) used to solve structure: *SHELXS97* (Sheldrick, 2008 ▶); program(s) used to refine structure: *SHELXL97* (Sheldrick, 2008 ▶); molecular graphics: *SHELXTL* (Sheldrick, 2008 ▶); software used to prepare material for publication: *SHELXL97*.

## Supplementary Material

Crystal structure: contains datablock(s) global, I. DOI: 10.1107/S1600536811034222/si2371sup1.cif

Structure factors: contains datablock(s) I. DOI: 10.1107/S1600536811034222/si2371Isup2.hkl

Additional supplementary materials:  crystallographic information; 3D view; checkCIF report

## Figures and Tables

**Table 1:** Selected bond lengths (Å)

Ir1—C18	1.995 (3)
Ir1—C1	1.999 (3)
Ir1—N2	2.067 (2)
Ir1—N1	2.091 (2)
Ir1—O4	2.1407 (18)
Ir1—O3	2.1414 (19)

**Fig. 1.**
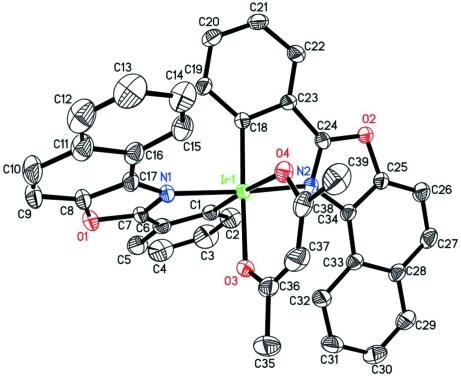
A view of the title complex. Displacement ellipsoids are drawn at the 30% probability level. H atoms omitted for clarity.

## supplementary crystallographic information

### Comment

According to the study of Lamansky's group in 2001 (Lamansky *et al.*, 2001), the luminous wavelength of complexes would change as the the conjugated system of (C—N) changed. Therefore, the arylnaphthoxazoles ligand was choosed to regulate luminous wavelength of phosphorescent materials, leading to get better electrophosphorescent materials.

The title complex (I) is a mononuclear iridium(III) complex (Fig. 1), in which the environment around the Ir^III^ ion is a distorted octahedral coordination geometry, the coordination conformation of the C, N and O atoms of the ligands adopt the *cis*-, *trans*- and *cis*- respectively, which is consistent with the similar reported complexes (Lamansky *et al.*, 2001). It can be illustrated (Fig. 1) that the carbon-metal bond is formed between the Ir^III^ ion and the carbon atom on the benzene ring rather than the C atom on the naphthalene ring. Moreover, there are two five-membered rings formed with (Ir1 C1 C6 C7 N1) and (Ir1 C18 C23 C24 N2), the average deviation of which are 0.0629 Å and 0.0719 Å, and the dihedral angle with their adjacent benzene rings (C1 C2 C3 C4 C5 C6) and (C18 C19 C20 C21 C22 C23) are 6.5 (2)° and 6.9 (1)° respectively, and the dihedral angle with their adjacent oxazole heterocycle (N1 O1 C7–17) and (N2 O2 C24–34) are 9.2 (1)° and 11.4 (1)° respectively. It shows from the Table 1 that the increase of the bond distance from Ir—C to Ir—N and Ir—O is caused by the increase of the covalent component between the coordination atoms from C to N and O of which the electronegativity decreases gradually.

### Experimental

The ligand 2-arylnaphth[1,2-*d*]oxazole was prepared according to the literature (Abbady, 1979). The ligand (0.54 gram, 2.2 mmol) and IrCl_3_.3H_2_O (0.35 gram, 1 mmol) were added to 20 ml 2-ethoxyethanol: H_2_O(3:1, *v*/*v*) solution under inert gas atmosphere at 120 °C for 24 h, and then the intermediate product, acetylacetonate(10 ml) and Na_2_CO_3_ (1.06 gram, 10 mmol) were refluxed for 12 h. After cooling to room temperature, the colored precipitate was then filtered and washed with ethanol and water. The crude product was flash chromatographed using a silica/dichloromethane column to yield *ca*. 58% of the pure title compound after solvent evaporation and drying.

### Refinement

H atoms were positioned geometrically and refined using a riding model with C—H =0.93–0.96 Å and with *U*_iso_(H) = 1.2 (1.5 for methyl groups) times *U*_eq_(C).

### Crystal data

**Table:**

[Ir(C_17_H_10_NO)_2_(C_5_H_7_O_2_)]	*F*(000) = 3072
*M**_r_* = 779.83	*D*_x_ = 1.675 Mg m^−^^3^
Monoclinic, *C*2/*c*	Mo *K*α radiation, λ = 0.71073 Å
*a* = 16.640 (3) Å	Cell parameters from 198 reflections
*b* = 17.384 (3) Å	θ = 2.5–26.0°
*c* = 21.461 (4) Å	µ = 4.36 mm^−^^1^
β = 95.026 (2)°	*T* = 273 K
*V* = 6184.3 (19) Å^3^	Prismatic, yellow
*Z* = 8	0.30 × 0.20 × 0.20 mm

### Data collection

**Table:**

Bruker SMART CCD area-detector diffractometer	7558 independent reflections
Radiation source: fine-focus sealed tube	6091 reflections with *I* > 2σ(*I*)
graphite	*R*_int_ = 0.031
phi and ω scans	θ_max_ = 28.2°, θ_min_ = 2.3°
Absorption correction: multi-scan (*SADABS*; Sheldrick, 2004)	*h* = −22→22
*T*_min_ = 0.354, *T*_max_ = 0.476	*k* = −23→22
28178 measured reflections	*l* = −28→28

### Refinement

**Table:**

Refinement on *F*^2^	Primary atom site location: structure-invariant direct methods
Least-squares matrix: full	Secondary atom site location: difference Fourier map
*R*[*F*^2^ > 2σ(*F*^2^)] = 0.023	Hydrogen site location: inferred from neighbouring sites
*wR*(*F*^2^) = 0.051	H-atom parameters constrained
*S* = 1.01	*w* = 1/[σ^2^(*F*_o_^2^) + (0.0233*P*)^2^ + 0.650*P*] where *P* = (*F*_o_^2^ + 2*F*_c_^2^)/3
7558 reflections	(Δ/σ)_max_ = 0.003
415 parameters	Δρ_max_ = 0.56 e Å^−^^3^
0 restraints	Δρ_min_ = −0.31 e Å^−^^3^

### Special details

**Table:**

Geometry. All e.s.d.'s (except the e.s.d. in the dihedral angle between two l.s. planes) are estimated using the full covariance matrix. The cell e.s.d.'s are taken into account individually in the estimation of e.s.d.'s in distances, angles and torsion angles; correlations between e.s.d.'s in cell parameters are only used when they are defined by crystal symmetry. An approximate (isotropic) treatment of cell e.s.d.'s is used for estimating e.s.d.'s involving l.s. planes.
Refinement. Refinement of *F*^2^ against ALL reflections. The weighted *R*-factor *wR* and goodness of fit *S* are based on *F*^2^, conventional *R*-factors *R* are based on *F*, with *F* set to zero for negative *F*^2^. The threshold expression of *F*^2^ > σ(*F*^2^) is used only for calculating *R*-factors(gt) *etc*. and is not relevant to the choice of reflections for refinement. *R*-factors based on *F*^2^ are statistically about twice as large as those based on *F*, and *R*- factors based on ALL data will be even larger.

### Fractional atomic coordinates and isotropic or equivalent isotropic displacement parameters (Å^2^)

**Table:**

	*x*	*y*	*z*	*U*_iso_*/*U*_eq_	
Ir1	0.197297 (6)	0.080046 (6)	0.624868 (4)	0.03071 (4)	
C1	0.11803 (17)	0.16584 (16)	0.60964 (13)	0.0388 (6)	
O1	0.18897 (14)	0.19239 (12)	0.45599 (10)	0.0566 (6)	
N1	0.21509 (14)	0.10506 (14)	0.53177 (10)	0.0375 (5)	
N2	0.16297 (13)	0.05731 (12)	0.71327 (10)	0.0325 (5)	
C2	0.0616 (2)	0.19443 (19)	0.64860 (17)	0.0565 (9)	
H2A	0.0531	0.1682	0.6852	0.068*	
O2	0.09828 (12)	−0.02524 (11)	0.77095 (8)	0.0434 (5)	
C3	0.0187 (2)	0.2604 (2)	0.6338 (2)	0.0770 (11)	
H3A	−0.0175	0.2785	0.6610	0.092*	
O3	0.29032 (12)	0.16332 (11)	0.64681 (9)	0.0441 (5)	
C4	0.0280 (2)	0.3008 (2)	0.5787 (2)	0.0773 (12)	
H4A	−0.0010	0.3457	0.5698	0.093*	
O4	0.28637 (11)	−0.00711 (11)	0.64615 (9)	0.0416 (5)	
C5	0.0802 (2)	0.27381 (19)	0.53807 (17)	0.0624 (10)	
H5A	0.0864	0.2995	0.5008	0.075*	
C6	0.12409 (18)	0.20665 (16)	0.55357 (14)	0.0448 (7)	
C7	0.17719 (18)	0.16926 (16)	0.51483 (13)	0.0419 (7)	
C8	0.2376 (2)	0.1358 (2)	0.43351 (14)	0.0529 (8)	
C9	0.2651 (3)	0.1344 (3)	0.37428 (16)	0.0720 (11)	
H9A	0.2533	0.1736	0.3454	0.086*	
C10	0.3104 (3)	0.0726 (3)	0.36111 (17)	0.0795 (14)	
H10A	0.3304	0.0696	0.3221	0.095*	
C11	0.3284 (2)	0.0119 (2)	0.40499 (16)	0.0637 (10)	
C12	0.3728 (3)	−0.0527 (3)	0.3898 (2)	0.0865 (13)	
H12A	0.3926	−0.0551	0.3506	0.104*	
C13	0.3878 (3)	−0.1112 (3)	0.4295 (2)	0.0875 (13)	
H13A	0.4182	−0.1529	0.4180	0.105*	
C14	0.3575 (2)	−0.1100 (2)	0.48947 (18)	0.0706 (10)	
H14A	0.3668	−0.1514	0.5166	0.085*	
C15	0.31459 (19)	−0.0478 (2)	0.50718 (15)	0.0550 (8)	
H15A	0.2949	−0.0470	0.5464	0.066*	
C16	0.30009 (18)	0.0147 (2)	0.46627 (14)	0.0486 (8)	
C17	0.25393 (18)	0.08108 (18)	0.47913 (13)	0.0443 (7)	
C18	0.11811 (15)	−0.00436 (15)	0.60453 (12)	0.0318 (6)	
C19	0.08870 (17)	−0.03322 (16)	0.54606 (13)	0.0402 (7)	
H19A	0.1014	−0.0080	0.5100	0.048*	
C20	0.04120 (19)	−0.09833 (18)	0.54086 (15)	0.0486 (8)	
H20A	0.0228	−0.1163	0.5013	0.058*	
C21	0.02032 (19)	−0.13742 (18)	0.59310 (14)	0.0498 (8)	
H21A	−0.0114	−0.1815	0.5886	0.060*	
C22	0.04648 (18)	−0.11104 (17)	0.65172 (14)	0.0448 (7)	
H22A	0.0324	−0.1366	0.6873	0.054*	
C23	0.09442 (16)	−0.04538 (16)	0.65690 (12)	0.0352 (6)	
C24	0.12111 (16)	−0.00633 (16)	0.71406 (12)	0.0356 (6)	
C25	0.12748 (17)	0.03448 (17)	0.80972 (13)	0.0403 (7)	
C26	0.11666 (19)	0.0415 (2)	0.87263 (14)	0.0523 (8)	
H26A	0.0891	0.0048	0.8940	0.063*	
C27	0.1491 (2)	0.1060 (2)	0.90122 (14)	0.0536 (8)	
H27A	0.1454	0.1124	0.9439	0.064*	
C28	0.18800 (19)	0.16318 (18)	0.86820 (13)	0.0455 (7)	
C29	0.2188 (2)	0.2304 (2)	0.89932 (15)	0.0609 (9)	
H29A	0.2150	0.2352	0.9421	0.073*	
C30	0.2537 (3)	0.2877 (2)	0.86859 (17)	0.0711 (11)	
H30A	0.2732	0.3313	0.8901	0.085*	
C31	0.2603 (2)	0.2809 (2)	0.80412 (17)	0.0695 (11)	
H31A	0.2838	0.3205	0.7830	0.083*	
C32	0.2326 (2)	0.21693 (17)	0.77178 (15)	0.0536 (8)	
H32A	0.2370	0.2137	0.7290	0.064*	
C33	0.19741 (17)	0.15592 (16)	0.80297 (13)	0.0389 (6)	
C34	0.16667 (17)	0.08667 (15)	0.77478 (12)	0.0365 (6)	
C35	0.4142 (2)	0.2183 (2)	0.68251 (19)	0.0828 (13)	
H35A	0.3834	0.2640	0.6726	0.124*	
H35B	0.4601	0.2170	0.6583	0.124*	
H35C	0.4323	0.2182	0.7262	0.124*	
C36	0.3621 (2)	0.1482 (2)	0.66736 (14)	0.0534 (8)	
C37	0.3956 (2)	0.0755 (2)	0.67846 (18)	0.0658 (10)	
H37A	0.4491	0.0744	0.6951	0.079*	
C38	0.35889 (19)	0.0046 (2)	0.66777 (14)	0.0520 (8)	
C39	0.4084 (2)	−0.0675 (2)	0.6810 (2)	0.0802 (13)	
H39A	0.3752	−0.1119	0.6718	0.120*	
H39B	0.4286	−0.0684	0.7243	0.120*	
H39C	0.4529	−0.0679	0.6554	0.120*	

### Atomic displacement parameters (Å^2^)

**Table:**

	*U*^11^	*U*^22^	*U*^33^	*U*^12^	*U*^13^	*U*^23^
Ir1	0.03189 (6)	0.03227 (6)	0.02784 (6)	−0.00094 (5)	0.00197 (4)	0.00314 (5)
C1	0.0385 (16)	0.0343 (15)	0.0421 (16)	−0.0019 (12)	−0.0046 (13)	−0.0013 (12)
O1	0.0761 (16)	0.0517 (14)	0.0404 (12)	−0.0120 (12)	−0.0034 (11)	0.0176 (10)
N1	0.0408 (13)	0.0411 (13)	0.0303 (12)	−0.0056 (11)	0.0014 (10)	0.0065 (10)
N2	0.0385 (13)	0.0335 (12)	0.0258 (11)	−0.0027 (10)	0.0046 (9)	−0.0005 (9)
C2	0.054 (2)	0.052 (2)	0.064 (2)	0.0109 (16)	0.0053 (17)	−0.0024 (17)
O2	0.0557 (13)	0.0450 (12)	0.0295 (10)	−0.0139 (10)	0.0050 (9)	0.0025 (9)
C3	0.065 (2)	0.070 (3)	0.094 (3)	0.026 (2)	−0.002 (2)	−0.008 (2)
O3	0.0455 (12)	0.0467 (12)	0.0394 (11)	−0.0140 (10)	−0.0004 (9)	0.0069 (9)
C4	0.073 (3)	0.053 (2)	0.100 (3)	0.027 (2)	−0.023 (2)	−0.002 (2)
O4	0.0412 (12)	0.0449 (12)	0.0385 (11)	0.0093 (9)	0.0020 (9)	0.0021 (9)
C5	0.074 (2)	0.0420 (19)	0.066 (2)	0.0046 (18)	−0.0234 (19)	0.0066 (17)
C6	0.0493 (18)	0.0336 (16)	0.0484 (18)	−0.0040 (13)	−0.0136 (14)	0.0029 (13)
C7	0.0508 (18)	0.0383 (16)	0.0345 (15)	−0.0122 (14)	−0.0079 (13)	0.0108 (13)
C8	0.063 (2)	0.061 (2)	0.0347 (17)	−0.0193 (18)	0.0013 (15)	0.0124 (15)
C9	0.090 (3)	0.088 (3)	0.039 (2)	−0.027 (3)	0.0094 (19)	0.0187 (19)
C10	0.093 (3)	0.109 (4)	0.041 (2)	−0.021 (3)	0.027 (2)	0.006 (2)
C11	0.057 (2)	0.089 (3)	0.047 (2)	−0.009 (2)	0.0195 (17)	−0.002 (2)
C12	0.071 (3)	0.129 (4)	0.064 (3)	−0.002 (3)	0.031 (2)	−0.015 (3)
C13	0.072 (3)	0.107 (4)	0.086 (3)	0.024 (3)	0.023 (2)	−0.022 (3)
C14	0.061 (2)	0.089 (3)	0.063 (2)	0.018 (2)	0.0055 (19)	−0.007 (2)
C15	0.052 (2)	0.070 (2)	0.0440 (19)	0.0086 (17)	0.0074 (15)	−0.0019 (17)
C16	0.0417 (17)	0.068 (2)	0.0368 (17)	−0.0079 (16)	0.0062 (13)	−0.0025 (15)
C17	0.0449 (17)	0.057 (2)	0.0308 (15)	−0.0135 (15)	0.0036 (13)	0.0051 (14)
C18	0.0317 (14)	0.0328 (14)	0.0311 (14)	0.0021 (11)	0.0037 (11)	0.0008 (11)
C19	0.0444 (17)	0.0457 (18)	0.0306 (15)	−0.0026 (14)	0.0035 (12)	−0.0024 (12)
C20	0.0502 (19)	0.0533 (19)	0.0422 (18)	−0.0090 (15)	0.0038 (14)	−0.0138 (14)
C21	0.057 (2)	0.0437 (18)	0.0497 (19)	−0.0163 (15)	0.0097 (16)	−0.0099 (15)
C22	0.0516 (18)	0.0416 (16)	0.0422 (17)	−0.0082 (15)	0.0104 (14)	0.0025 (14)
C23	0.0395 (15)	0.0332 (14)	0.0330 (15)	−0.0028 (12)	0.0037 (12)	−0.0024 (12)
C24	0.0378 (15)	0.0380 (15)	0.0317 (14)	−0.0023 (12)	0.0076 (12)	0.0029 (12)
C25	0.0449 (17)	0.0450 (17)	0.0308 (15)	−0.0050 (14)	0.0021 (13)	0.0023 (12)
C26	0.060 (2)	0.063 (2)	0.0340 (16)	−0.0106 (17)	0.0092 (15)	0.0010 (15)
C27	0.063 (2)	0.069 (2)	0.0288 (16)	−0.0030 (18)	0.0048 (15)	−0.0058 (15)
C28	0.0531 (19)	0.0510 (19)	0.0321 (15)	−0.0016 (15)	0.0022 (13)	−0.0078 (13)
C29	0.078 (2)	0.063 (2)	0.0411 (18)	−0.0058 (19)	0.0041 (17)	−0.0151 (17)
C30	0.091 (3)	0.055 (2)	0.066 (3)	−0.010 (2)	−0.002 (2)	−0.0232 (19)
C31	0.101 (3)	0.049 (2)	0.059 (2)	−0.025 (2)	0.012 (2)	−0.0079 (17)
C32	0.074 (2)	0.0442 (19)	0.0428 (18)	−0.0115 (17)	0.0079 (16)	−0.0044 (14)
C33	0.0417 (16)	0.0383 (16)	0.0366 (15)	0.0000 (13)	0.0023 (13)	−0.0052 (12)
C34	0.0399 (15)	0.0401 (16)	0.0295 (14)	−0.0014 (12)	0.0033 (12)	−0.0024 (12)
C35	0.062 (2)	0.089 (3)	0.094 (3)	−0.037 (2)	−0.013 (2)	0.014 (2)
C36	0.0443 (19)	0.072 (2)	0.0435 (18)	−0.0193 (17)	−0.0001 (15)	0.0033 (16)
C37	0.0383 (18)	0.084 (3)	0.072 (3)	−0.0019 (19)	−0.0118 (17)	−0.002 (2)
C38	0.0433 (18)	0.072 (2)	0.0403 (18)	0.0143 (17)	0.0003 (14)	−0.0005 (16)
C39	0.063 (2)	0.086 (3)	0.088 (3)	0.035 (2)	−0.015 (2)	−0.005 (2)

### Geometric parameters (Å, °)

**Table:**

Ir1—C18	1.995 (3)	C15—H15A	0.9300
Ir1—C1	1.999 (3)	C16—C17	1.427 (4)
Ir1—N2	2.067 (2)	C18—C19	1.399 (4)
Ir1—N1	2.091 (2)	C18—C23	1.416 (3)
Ir1—O4	2.1407 (18)	C19—C20	1.379 (4)
Ir1—O3	2.1414 (19)	C19—H19A	0.9300
C1—C2	1.401 (4)	C20—C21	1.381 (4)
C1—C6	1.408 (4)	C20—H20A	0.9300
O1—C7	1.356 (3)	C21—C22	1.374 (4)
O1—C8	1.387 (4)	C21—H21A	0.9300
N1—C7	1.317 (3)	C22—C23	1.391 (4)
N1—C17	1.413 (4)	C22—H22A	0.9300
N2—C24	1.308 (3)	C23—C24	1.438 (4)
N2—C34	1.412 (3)	C25—C34	1.377 (4)
C2—C3	1.374 (5)	C25—C26	1.383 (4)
C2—H2A	0.9300	C26—C27	1.365 (5)
O2—C24	1.351 (3)	C26—H26A	0.9300
O2—C25	1.391 (3)	C27—C28	1.411 (4)
C3—C4	1.394 (5)	C27—H27A	0.9300
C3—H3A	0.9300	C28—C29	1.420 (4)
O3—C36	1.264 (4)	C28—C33	1.428 (4)
C4—C5	1.366 (5)	C29—C30	1.354 (5)
C4—H4A	0.9300	C29—H29A	0.9300
O4—C38	1.271 (3)	C30—C31	1.402 (5)
C5—C6	1.402 (4)	C30—H30A	0.9300
C5—H5A	0.9300	C31—C32	1.369 (4)
C6—C7	1.422 (4)	C31—H31A	0.9300
C8—C17	1.375 (4)	C32—C33	1.409 (4)
C8—C9	1.388 (4)	C32—H32A	0.9300
C9—C10	1.356 (6)	C33—C34	1.422 (4)
C9—H9A	0.9300	C35—C36	1.513 (4)
C10—C11	1.428 (5)	C35—H35A	0.9600
C10—H10A	0.9300	C35—H35B	0.9600
C11—C12	1.399 (5)	C35—H35C	0.9600
C11—C16	1.436 (4)	C36—C37	1.394 (5)
C12—C13	1.337 (6)	C37—C38	1.385 (5)
C12—H12A	0.9300	C37—H37A	0.9300
C13—C14	1.422 (5)	C38—C39	1.513 (4)
C13—H13A	0.9300	C39—H39A	0.9600
C14—C15	1.367 (5)	C39—H39B	0.9600
C14—H14A	0.9300	C39—H39C	0.9600
C15—C16	1.405 (4)		
			
C18—Ir1—C1	95.70 (11)	C8—C17—C16	119.9 (3)
C18—Ir1—N2	80.20 (9)	N1—C17—C16	133.3 (3)
C1—Ir1—N2	93.51 (10)	C19—C18—C23	115.6 (2)
C18—Ir1—N1	95.17 (10)	C19—C18—Ir1	129.2 (2)
C1—Ir1—N1	80.52 (10)	C23—C18—Ir1	114.92 (19)
N2—Ir1—N1	172.10 (8)	C20—C19—C18	121.3 (3)
C18—Ir1—O4	87.58 (9)	C20—C19—H19A	119.3
C1—Ir1—O4	176.06 (9)	C18—C19—H19A	119.3
N2—Ir1—O4	84.87 (8)	C19—C20—C21	121.4 (3)
N1—Ir1—O4	101.40 (8)	C19—C20—H20A	119.3
C18—Ir1—O3	175.06 (9)	C21—C20—H20A	119.3
C1—Ir1—O3	89.17 (10)	C22—C21—C20	119.8 (3)
N2—Ir1—O3	100.37 (8)	C22—C21—H21A	120.1
N1—Ir1—O3	84.80 (8)	C20—C21—H21A	120.1
O4—Ir1—O3	87.59 (8)	C21—C22—C23	118.7 (3)
C2—C1—C6	115.6 (3)	C21—C22—H22A	120.6
C2—C1—Ir1	129.3 (2)	C23—C22—H22A	120.6
C6—C1—Ir1	114.8 (2)	C22—C23—C18	123.2 (2)
C7—O1—C8	104.5 (2)	C22—C23—C24	126.0 (2)
C7—N1—C17	105.7 (2)	C18—C23—C24	110.7 (2)
C7—N1—Ir1	109.60 (19)	N2—C24—O2	114.2 (2)
C17—N1—Ir1	144.7 (2)	N2—C24—C23	120.9 (2)
C24—N2—C34	105.9 (2)	O2—C24—C23	124.5 (2)
C24—N2—Ir1	111.34 (17)	C34—C25—C26	125.6 (3)
C34—N2—Ir1	142.78 (18)	C34—C25—O2	108.9 (2)
C3—C2—C1	121.3 (3)	C26—C25—O2	125.5 (3)
C3—C2—H2A	119.3	C27—C26—C25	115.5 (3)
C1—C2—H2A	119.3	C27—C26—H26A	122.2
C24—O2—C25	104.3 (2)	C25—C26—H26A	122.2
C2—C3—C4	121.5 (4)	C26—C27—C28	122.2 (3)
C2—C3—H3A	119.3	C26—C27—H27A	118.9
C4—C3—H3A	119.3	C28—C27—H27A	118.9
C36—O3—Ir1	125.4 (2)	C27—C28—C29	120.5 (3)
C5—C4—C3	119.5 (3)	C27—C28—C33	121.6 (3)
C5—C4—H4A	120.2	C29—C28—C33	117.9 (3)
C3—C4—H4A	120.2	C30—C29—C28	122.0 (3)
C38—O4—Ir1	125.6 (2)	C30—C29—H29A	119.0
C4—C5—C6	118.7 (3)	C28—C29—H29A	119.0
C4—C5—H5A	120.6	C29—C30—C31	119.5 (3)
C6—C5—H5A	120.6	C29—C30—H30A	120.3
C5—C6—C1	123.2 (3)	C31—C30—H30A	120.3
C5—C6—C7	125.4 (3)	C32—C31—C30	121.2 (3)
C1—C6—C7	111.3 (3)	C32—C31—H31A	119.4
N1—C7—O1	113.8 (3)	C30—C31—H31A	119.4
N1—C7—C6	122.2 (3)	C31—C32—C33	120.4 (3)
O1—C7—C6	123.8 (3)	C31—C32—H32A	119.8
C17—C8—O1	109.1 (3)	C33—C32—H32A	119.8
C17—C8—C9	125.5 (4)	C32—C33—C34	125.7 (3)
O1—C8—C9	125.4 (3)	C32—C33—C28	119.0 (3)
C10—C9—C8	116.0 (4)	C34—C33—C28	115.2 (3)
C10—C9—H9A	122.0	C25—C34—N2	106.7 (2)
C8—C9—H9A	122.0	C25—C34—C33	119.7 (3)
C9—C10—C11	122.4 (3)	N2—C34—C33	133.5 (2)
C9—C10—H10A	118.8	C36—C35—H35A	109.5
C11—C10—H10A	118.8	C36—C35—H35B	109.5
C12—C11—C10	121.6 (4)	H35A—C35—H35B	109.5
C12—C11—C16	117.6 (4)	C36—C35—H35C	109.5
C10—C11—C16	120.8 (4)	H35A—C35—H35C	109.5
C13—C12—C11	122.4 (4)	H35B—C35—H35C	109.5
C13—C12—H12A	118.8	O3—C36—C37	126.8 (3)
C11—C12—H12A	118.8	O3—C36—C35	114.5 (3)
C12—C13—C14	120.3 (4)	C37—C36—C35	118.7 (3)
C12—C13—H13A	119.9	C38—C37—C36	128.0 (3)
C14—C13—H13A	119.9	C38—C37—H37A	116.0
C15—C14—C13	119.9 (4)	C36—C37—H37A	116.0
C15—C14—H14A	120.0	O4—C38—C37	126.4 (3)
C13—C14—H14A	120.0	O4—C38—C39	114.8 (3)
C14—C15—C16	120.2 (3)	C37—C38—C39	118.7 (3)
C14—C15—H15A	119.9	C38—C39—H39A	109.5
C16—C15—H15A	119.9	C38—C39—H39B	109.5
C15—C16—C17	124.8 (3)	H39A—C39—H39B	109.5
C15—C16—C11	119.6 (3)	C38—C39—H39C	109.5
C17—C16—C11	115.4 (3)	H39A—C39—H39C	109.5
C8—C17—N1	106.8 (3)	H39B—C39—H39C	109.5
			
C18—Ir1—C1—C2	79.9 (3)	C9—C8—C17—N1	179.5 (3)
N2—Ir1—C1—C2	−0.6 (3)	O1—C8—C17—C16	177.1 (3)
N1—Ir1—C1—C2	174.2 (3)	C9—C8—C17—C16	−2.6 (5)
O4—Ir1—C1—C2	−66.3 (15)	C7—N1—C17—C8	1.7 (3)
O3—Ir1—C1—C2	−101.0 (3)	Ir1—N1—C17—C8	−176.1 (2)
C18—Ir1—C1—C6	−105.2 (2)	C7—N1—C17—C16	−175.9 (3)
N2—Ir1—C1—C6	174.3 (2)	Ir1—N1—C17—C16	6.4 (6)
N1—Ir1—C1—C6	−10.9 (2)	C15—C16—C17—C8	−174.3 (3)
O4—Ir1—C1—C6	108.6 (14)	C11—C16—C17—C8	1.7 (4)
O3—Ir1—C1—C6	73.9 (2)	C15—C16—C17—N1	3.0 (5)
C18—Ir1—N1—C7	104.92 (19)	C11—C16—C17—N1	179.0 (3)
C1—Ir1—N1—C7	9.99 (19)	C1—Ir1—C18—C19	81.4 (3)
N2—Ir1—N1—C7	51.2 (7)	N2—Ir1—C18—C19	174.0 (3)
O4—Ir1—N1—C7	−166.51 (18)	N1—Ir1—C18—C19	0.4 (3)
O3—Ir1—N1—C7	−80.04 (19)	O4—Ir1—C18—C19	−100.8 (3)
C18—Ir1—N1—C17	−77.4 (3)	O3—Ir1—C18—C19	−89.0 (10)
C1—Ir1—N1—C17	−172.3 (3)	C1—Ir1—C18—C23	−105.3 (2)
N2—Ir1—N1—C17	−131.1 (6)	N2—Ir1—C18—C23	−12.70 (19)
O4—Ir1—N1—C17	11.2 (3)	N1—Ir1—C18—C23	173.7 (2)
O3—Ir1—N1—C17	97.7 (3)	O4—Ir1—C18—C23	72.5 (2)
C18—Ir1—N2—C24	11.46 (19)	O3—Ir1—C18—C23	84.4 (10)
C1—Ir1—N2—C24	106.6 (2)	C23—C18—C19—C20	−1.3 (4)
N1—Ir1—N2—C24	66.0 (7)	Ir1—C18—C19—C20	172.0 (2)
O4—Ir1—N2—C24	−76.97 (19)	C18—C19—C20—C21	0.4 (5)
O3—Ir1—N2—C24	−163.56 (18)	C19—C20—C21—C22	0.6 (5)
C18—Ir1—N2—C34	−166.7 (3)	C20—C21—C22—C23	−0.6 (5)
C1—Ir1—N2—C34	−71.5 (3)	C21—C22—C23—C18	−0.3 (5)
N1—Ir1—N2—C34	−112.1 (6)	C21—C22—C23—C24	174.3 (3)
O4—Ir1—N2—C34	104.9 (3)	C19—C18—C23—C22	1.3 (4)
O3—Ir1—N2—C34	18.3 (3)	Ir1—C18—C23—C22	−173.0 (2)
C6—C1—C2—C3	−3.2 (5)	C19—C18—C23—C24	−174.1 (2)
Ir1—C1—C2—C3	171.6 (3)	Ir1—C18—C23—C24	11.6 (3)
C1—C2—C3—C4	1.4 (6)	C34—N2—C24—O2	−2.5 (3)
C18—Ir1—O3—C36	−15.9 (11)	Ir1—N2—C24—O2	178.68 (17)
C1—Ir1—O3—C36	173.7 (2)	C34—N2—C24—C23	170.1 (2)
N2—Ir1—O3—C36	80.3 (2)	Ir1—N2—C24—C23	−8.8 (3)
N1—Ir1—O3—C36	−105.8 (2)	C25—O2—C24—N2	1.7 (3)
O4—Ir1—O3—C36	−4.1 (2)	C25—O2—C24—C23	−170.6 (3)
C2—C3—C4—C5	0.9 (6)	C22—C23—C24—N2	−176.7 (3)
C18—Ir1—O4—C38	−175.9 (2)	C18—C23—C24—N2	−1.5 (4)
C1—Ir1—O4—C38	−29.5 (15)	C22—C23—C24—O2	−4.9 (5)
N2—Ir1—O4—C38	−95.5 (2)	C18—C23—C24—O2	170.3 (2)
N1—Ir1—O4—C38	89.4 (2)	C24—O2—C25—C34	−0.1 (3)
O3—Ir1—O4—C38	5.2 (2)	C24—O2—C25—C26	178.0 (3)
C3—C4—C5—C6	−1.1 (6)	C34—C25—C26—C27	−0.7 (5)
C4—C5—C6—C1	−0.9 (5)	O2—C25—C26—C27	−178.5 (3)
C4—C5—C6—C7	176.4 (3)	C25—C26—C27—C28	2.6 (5)
C2—C1—C6—C5	3.0 (4)	C26—C27—C28—C29	178.2 (3)
Ir1—C1—C6—C5	−172.6 (2)	C26—C27—C28—C33	−1.5 (5)
C2—C1—C6—C7	−174.6 (3)	C27—C28—C29—C30	−177.4 (3)
Ir1—C1—C6—C7	9.7 (3)	C33—C28—C29—C30	2.3 (5)
C17—N1—C7—O1	−2.0 (3)	C28—C29—C30—C31	−0.3 (6)
Ir1—N1—C7—O1	176.59 (18)	C29—C30—C31—C32	−0.5 (6)
C17—N1—C7—C6	173.3 (3)	C30—C31—C32—C33	−0.6 (6)
Ir1—N1—C7—C6	−8.0 (3)	C31—C32—C33—C34	−179.8 (3)
C8—O1—C7—N1	1.5 (3)	C31—C32—C33—C28	2.6 (5)
C8—O1—C7—C6	−173.7 (3)	C27—C28—C33—C32	176.3 (3)
C5—C6—C7—N1	−178.3 (3)	C29—C28—C33—C32	−3.4 (5)
C1—C6—C7—N1	−0.7 (4)	C27—C28—C33—C34	−1.5 (4)
C5—C6—C7—O1	−3.4 (5)	C29—C28—C33—C34	178.8 (3)
C1—C6—C7—O1	174.2 (2)	C26—C25—C34—N2	−179.4 (3)
C7—O1—C8—C17	−0.4 (3)	O2—C25—C34—N2	−1.3 (3)
C7—O1—C8—C9	179.4 (3)	C26—C25—C34—C33	−2.3 (5)
C17—C8—C9—C10	1.4 (6)	O2—C25—C34—C33	175.8 (2)
O1—C8—C9—C10	−178.3 (3)	C24—N2—C34—C25	2.3 (3)
C8—C9—C10—C11	0.5 (6)	Ir1—N2—C34—C25	−179.5 (2)
C9—C10—C11—C12	177.8 (4)	C24—N2—C34—C33	−174.3 (3)
C9—C10—C11—C16	−1.2 (6)	Ir1—N2—C34—C33	3.9 (5)
C10—C11—C12—C13	−177.7 (4)	C32—C33—C34—C25	−174.4 (3)
C16—C11—C12—C13	1.4 (6)	C28—C33—C34—C25	3.2 (4)
C11—C12—C13—C14	0.8 (7)	C32—C33—C34—N2	1.8 (5)
C12—C13—C14—C15	−1.7 (7)	C28—C33—C34—N2	179.4 (3)
C13—C14—C15—C16	0.2 (6)	Ir1—O3—C36—C37	1.6 (5)
C14—C15—C16—C17	177.8 (3)	Ir1—O3—C36—C35	−176.6 (2)
C14—C15—C16—C11	2.0 (5)	O3—C36—C37—C38	2.2 (6)
C12—C11—C16—C15	−2.8 (5)	C35—C36—C37—C38	−179.7 (4)
C10—C11—C16—C15	176.3 (3)	Ir1—O4—C38—C37	−3.8 (5)
C12—C11—C16—C17	−179.0 (3)	Ir1—O4—C38—C39	177.7 (2)
C10—C11—C16—C17	0.1 (5)	C36—C37—C38—O4	−0.9 (6)
O1—C8—C17—N1	−0.8 (3)	C36—C37—C38—C39	177.5 (4)
